# "When they are all grown, I will tell them”: Experience and perceptions of parental self-disclosure of HIV status to children in Nairobi, Kenya

**DOI:** 10.1186/s12889-023-15387-3

**Published:** 2023-03-17

**Authors:** Cyrus Mugo, Olivia Firdawsi, Jiayu Wang, Irene N. Njuguna, Dalton C. Wamalwa, Jennifer A. Slyker, Grace C. John-Stewart, Gabrielle O’Malley, Anjuli D. Wagner

**Affiliations:** 1 Department of Research and Programs, Kenyatta National Hospital, Nairobi, 00202 Kenya; 2 Department of Epidemiology, University of Washington, Box 359909, 3980 15th Ave. NE, Seattle, WA 98195 USA; 3 Department of Global Health, University of Washington, Box 359909, 3980 15th Ave. NE, Seattle, WA 98195 USA; 4 Department of Medicine, University of Washington, Box 359909, WA 98104 Seattle, USA; 5 Department of Medicine, University of Washington, Box 359909, 3980 15th Ave. NE, Seattle, WA 98195 USA; 6 Department of Pediatrics, University of Nairobi, Nairobi, KE 00202 USA

**Keywords:** HIV, Sub-Saharan Africa, Self-disclosure, Parental disclosure, HIV stigma, Children

## Abstract

**Background:**

There is mixed evidence on the influence of self-disclosure of one’s HIV status on mental health, health behaviours and clinical outcomes. We studied the patterns of self-disclosure among parents living with HIV, and factors that influence parental disclosure.

**Methods:**

This mixed-methods study was among adults in HIV care participating in a study assessing the uptake of pediatric index-case testing. They completed a survey to provide demographic and HIV-related health information, and assess self-disclosure to partners, children and others. We ran generalized linear models to determine factors associated with disclosure and reported prevalence ratios (PR). Eighteen participants also participated in in-depth interviews to explore perceived barriers and facilitators of self-disclosure to one’s child. A content analysis approach was used to analyze interview transcripts.

**Results:**

Of 493 caregivers, 238 (48%) had a child ≥ 6 years old who could potentially be disclosed to about their parent's HIV status. Of 238 participants, 205 (86%) were female, median age was 35 years, and 132 (55%) were in a stable relationship. Among those in a stable relationship, 96 (73%) knew their partner’s HIV status, with 79 (60%) reporting that their partner was living with HIV. Caregivers had known their HIV status for a median 2 years, and the median age of their oldest child was 11 years old. Older caregiver age and older first born child’s age were each associated with 10% higher likelihood of having disclosed to a child (PR: 1.10 [1.06–1.13] and PR: 1.10 [1.06–1.15], per year of age, respectively).

The child’s age or perceived maturity and fear of causing anxiety to the child inhibited disclosure. Child’s sexual activity was a motivator for disclosure, as well as the belief that disclosing was the “right thing to do”. Caregivers advocated for peer and counseling support to gain insight on appropriate ways to disclose their status.

**Conclusions:**

Child’s age is a key consideration for parents to disclose their own HIV status to their children. While parents were open to disclosing their HIV status to their children, there is a need to address barriers including anticipated stigma, and fear that disclosure will cause distress to their children.

## Introduction

Disclosure of one’s HIV status (self-disclosure) [1] to family members, partners and others is associated with greater access to social support, improved antiretroviral therapy (ART) adherence, and viral suppression [2–4]. However, the evidence on the effect of disclosure on mental health is mixed with some studies showing improved esteem, emotional relief and reduced depressive symptoms, and others linking disclosure with elevated anxiety, depressive symptoms, stigma, and shame [2, 5–7], Recent literature particularly in developed countries highlight some of the benefits of parental disclosure to include improved communication within a family and quality of relationship and reduced parental stress. On the other hand, while most children received the information positively, some children experienced negative reactions including worry and anger [8]. Reported disclosure levels in sub-Saharan Africa range widely for disclosure to partners (16.7% to 86%) [9] and to family members (8% to 98%) [10]. However, fewer studies have reported on disclosure of parental HIV status to his/her children, particularly in sub-Saharan Africa [5, 11]. In a systematic review of global literature on parental self-disclosure, 4 of the 38 studies included were conducted in Africa, and the prevalence of parental HIV disclosure to children was low (11% to 50%) [5]. A recent study in Rwanda reported similarly high levels of non-disclosure (44%), and more explicitly showed some of the negative responses of parental disclosure by children, especially if full disclosure was not done, including loss of trust with parents and poorer mental health. There was, however, improved child resilience and mental health where structured full parental disclosure was done [11].

The differences in family structures, norms, and culture between people living with HIV from, for example, sub-Saharan Africa and high-income settings suggests that there may be differences in how self-disclosure is done, and its impact, especially on children [9, 12]. Countries like Kenya in the Eastern and Southern African regions have the highest burden of HIV among adults [13]. The high survival rates realized with increasing availability of highly efficacious antiretroviral therapy means that many persons living with HIV can start families and can raise children not living with HIV. [14]. This progress is blunted by the high prevalence of HIV stigma that remains a barrier to HIV disclosure, interpersonal relationships, and optimal HIV treatment behaviors and outcomes [15–18].

Parents may weigh many factors when deciding whether or not to disclose their HIV status to their children, including the child’s age and developmental level, presence of stigma in the family or community, and perceived benefits of disclosure to the child [5, 19]. Non-disclosing parents may go to great lengths to keep their HIV status secret and may jeopardize their own health (i.e. delaying taking medications or only scheduling medical appointments when children are absent) [20, 21] and report more symptoms of depression than those who have disclosed [21]. Parents living with HIV need supportive services to deal with the challenging and complex process of disclosing their HIV status to their children. Some interventions increased parental HIV status disclosure to their children [11, 22–24], although very few of the studies were conducted in the sub-Saharan African context [11, 22, 25]. To adapt these interventions for people living with HIV in sub-Saharan Africa, we must have a deeper understanding of the perspectives and needs of the intended audience.

We assessed the prevalence of disclosure of parental HIV status to children and compared to other disclosures (including to partners), and cofactors associated with disclosure to children in Kenya. We also explored experiences and perceptions of parents living with HIV disclosing their HIV status to their children.

## Methods

### Study design

This convergent parallel design mixed-methods study was nested within the *Counseling and Testing for Children at Home (CATCH)* study that was conducted in Kenya. The CATCH study collected both qualitative and quantitative data to assess the feasibility, acceptability and cost-effectiveness of a systematic offer of pediatric HIV testing to all adults enrolled in HIV care, with an option of conducting the test at home or in the clinic; the results of which have been published elsewhere [26–28].

### Recruitment and enrollment

Participants were adults living with HIV recruited from Kenyatta National Hospital (national referral hospital), 5 Nairobi City County facilities and 1 facility in Kisumu City County. The facilities were selected from a large number of HIV care facilities in the two counties due to their large population of adults living with HIV, and having participated in previous pediatric HIV treatment studies conducted by the research group. Unlike this study, however, the previous studies targeted children in inpatient facilities. Their parents, if in HIV care, represented a minority of adults in HIV care in these facilities. Caregivers were eligible to enroll in the study if they had at least one child 12 years of age or younger of unknown HIV status. To prevent inadvertent disclosure of the HIV status of the biological mother of any child, male caregivers recruited could only test their children after consent from the biological mother, or if the mother was not alive or at the time not a primary caregiver for the child. This resulted in over-representation of women in this study.

### Quantitative data collection and analysis

Data were collected between 2013 and 2016. At enrollment, study participants completed an enrollment questionnaire with demographic and HIV-related health information, including information on HIV disclosure. The questionnaire was administered by a research assistant in English, Swahili or Dholuo, and responses recorded on a phone or tablet through the open data kit (ODK) program. For this study, we included parents with at least one child who could potentially be disclosed to, estimated at ≥ 6 years old as recommended by the WHO HIV status disclosure guidelines [29]. Prevalence of disclosure to someone (partner, child, other family member, friend or other persons), partners (for those in a stable relationship or married), disclosure to children (for those with at least one child ≥ 6 years old) and disclosure to others (other family member other than a partner/child and friends) were calculated. For disclosure to the child, the 6 years of age cut off was selected since that is the recommended age to start parental HIV status disclosure [29]. We assessed statistical differences between the prevalences using chi-squared tests. To determine associations between disclosure to a child and socio-demographic and HIV-related factors, we ran generalized linear models with binomial family and log link. Factors assessed included age, gender, income, time since diagnosis, place of diagnosis, education level, knowledge of partner status, and partner HIV status. We reported prevalence ratios (PRs), 95% confidence intervals (95%CI) and p-values from Wald tests.

### Power calculations

We assessed the minimum detectable association (80% power at alpha = 0.05) between factors ranging between 10 to 50% frequency for outcomes of disclosure to children (14% prevalence). We had power to detect PRs greater than 2.40–3.67 for disclosure to a child. Analyses were conducted using R studio (Version 1.1.456, 2009–2018).

### Qualitative data collection and analysis

Participants who finished the quantitative study at Kenyatta National Hospital were recruited for the in-depth interview. The interviews, which were conducted in either English or Kiswahili, covered aspects of disclosure (covered in this paper); and cost and access to care, and perceptions regarding HIV testing (covered in a different paper) [30]. The recruitment continued until the interviewers noted saturation of information on the key themes. Interviews were conducted in a designated private room at the HIV clinic, were audio recorded, translated into English as needed, and transcribed. A content analysis approach was used to analyze interview transcripts [31]. A start list of codes was created from a socio-ecological conceptual framework developed by the authors based on existing parental disclosure literature to examine likely factors that affect parental disclosure of HIV, and subsequently mapped to a 2015 adaptation of the social ecological model applied to decision-making of parental HIV disclosure [32]. One transcript was independently coded by two analysts (OF and ADW). Applied codes were compared, discussed, and modified jointly by the analysts, and additional codes were added accordingly to the codebook. Each additional transcript was coded by primary coder (OF) and applied codes were reviewed by a secondary coder (ADW) with any disagreements noted and resolved through discussions. The coders used ATLAS.ti software (Scientific Software Developments, Berlin, Germany, 2012). The distribution of codes was then analyzed to identify themes as they emerged from the data [33].

### Mixed methods approach

Using primarily a parallel convergent design, we compared the qualitative and quantitative findings, noting areas of divergence or convergence. Additionally, the qualitative data provided a deeper explanation of the quantitative findings, particularly in relation to disclosure to children.

### Ethics statement

The CATCH study was approved by the University of Washington Institutional Review Board (IRB) and the Kenyatta National Hospital (KNH)/University of Nairobi (UoN) Ethics and Research Committee. Written informed consent was obtained from all participants in in-depth interviews. Oral consent was obtained from all participants completing the enrollment questionnaire.

## Results

Four hundred and ninety three caregivers completed the enrollment questionnaire, with 238 (48%) of those with a child ≥ 6 years old. Of the 238 participants, 205 (86%) were female, median age was 35 years, monthly household income was $68 USD (IQR: 43–145), which is approximately $2.3 USD per day, and 132 (55%) were in a stable relationship/married. Among those in a stable relationship/married, 96 (73%) knew their partner’s HIV status, with 79 (60%) reporting that their partner was living with HIV. Caregivers had known their HIV status for a median 2 years, and the median age of their oldest child was 11 years old (Table 1). The full study population from the parent study has been previously described [26, 27].

**Table 1:** Caregiver sociodemographic and HIV-related characteristics and their association with disclosure to a child

**Characteristics**	**Overall ** ***N*** ** = 238**	**No disclosure ** ***N*** ** = 206**	**Disclosure to a child ** ***N*** ** = 32**	**Prevalence ratio** ^1^	***p*** ** value (Wald test)**
	***Median (IQR)/ n(%)***	***Median (IQR)/ n(%)***	***Median (IQR)/ n(%)***	***PR (95% confidence interval)***	
Age (years)	35 (30–39)	34 (30–38)	43 (39–46)	**1.10 (1.07–1.13)**	** < 0.001**
Age of oldest child (years)	11 (8–14)	11 (8–14)	15 (10–20)	**1.10 (1.06–1.15)**	** < 0.001**
Gender					
Male	33 (14%)	27 (82%)	6 (18%)	Reference	
Female	205 (86%)	179 (87%)	26 (13%)	0.70 (0.29–1.69)	0.426
Income (USD)*					
≥ $2/day	101 (43%)	87 (86%)	14 (14%)	Reference	
< $2/day	135 (57%)	119 (88%)	16 (12%)	0.86 (0.42–1.75)	0.669
Years of education	9 (7–12)	9 (8–12)	8 (7–11)	0.94 (0.84–1.04)	0.201
Years since HIV diagnosis	2 (0–6)	2 (0–5)	4 (0–7)	1.07 (0.97–1.18)	0.160
Place of diagnosis					
VCT	68 (29%)	58 (85%)	10 (15%)	Reference	
PMTCT	61 (26%)	57 (93%)	4 (7%)	0.45 (0.14–1.42)	0.172
Hospital outpatient	62 (26%)	55 (89%)	7 (11%)	0.77 (0.29–2.02)	0.592
Hospital inpatient	41 (17%)	31 (76%)	10 (24%)	1.66 (0.69–3.98)	0.258
Home based testing	6 (2%)	5 (83%)	1 (17%)	1.13 (0.15–8.85)	0.905
Relationship status					
Currently not in a stable relationship	106 (45%)	94 (89%)	12 (11%)	Reference	
Currently in a stable relationship (married/ stable boyfriend or girlfriend)	132 (55%)	112 (85%)	20 (15%)	1.34 (0.65–2.74)	0.425
Partner HIV status, N = 132					
Unknown HIV status	36 (27%)	32 (89%)	4 (11%)	Reference	
Living with HIV	79 (60%)	64 (81%)	15 (19%)	1.71 (0.57–5.15)	0.341
Not living with HIV	17 (13%)	16 (94%)	1 (6%)	0.53 (0.06–4.74)	0.569

^1^Outcome: disclosure to a child

Bolded: Significant association (*p* < 0.05)

^*^Missing data due to lack of responses

All 238 participants had disclosed their HIV status to someone, 222 (93%) had disclosed to others (friend or family who was not a partner or their child), while 32 (13%) had disclosed their status to any of their children. Of the 132 who had a stable partner or were married, 124 (94%) had disclosed their status to their partner. The proportion that had disclosed to a child was significantly lower than the disclosure to a partner or other family/friend (*p* < 0.001 for both comparisons from a chi-squared test). Of the caregivers who had disclosed, their oldest child’s median age was 15 (IQR: 10–20) years. One year older caregiver age and one year older first born child’s age were each associated with 10% higher likelihood of having disclosed to a child (PR: 1.10 [95%CI: 1.06–1.13], *p* < 0.001 and PR: 1.10 [95%CI: 1.06–1.15], *p* < 0.001 per year of age, respectively). We found no evidence of associations between parental HIV status disclosure and other caregiver characteristics – gender, income, education, duration since HIV diagnosis, place of HIV diagnosis, relationship status, and partner HIV status (Table 1).

### Interviews

**Fig. 1:**
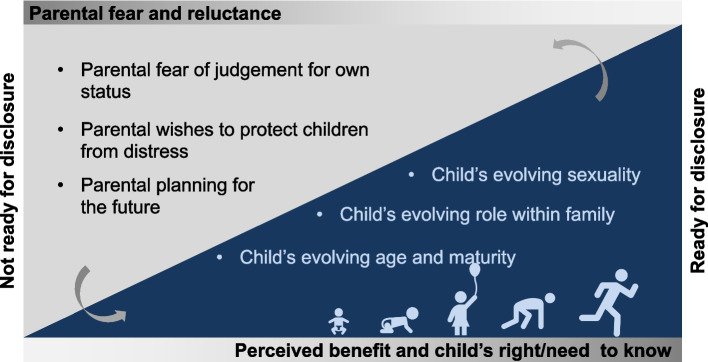
Child and parent readiness for disclosure

Eighteen participants subsequently completed in-depth interviews. The socio-demographic and HIV-related characteristics of those who completed the in-depth interviews mirrored the study population that completed the enrollment questionnaire [30]. Similar to results in the quantitative study where children’s age was a key factor determining parental disclosure, in considering disclosing to their children, interviewed parents highlighted a range of considerations related to child maturity and age, sexuality, and role within the family. They also highlighted parental fear of judgment for their own status, wishes to protect a child emotionally, and future parental care plans as other considerations (Table 2). Seventeen of 18 participants reported willingness to disclose their HIV status to their children in future. However, several participants reported a need for professional or peer assistance in the process. In Fig. 1, we depict some of the key factors identified in this study that may influence the decision by parents to disclose their HIV status to their children.

**Table 2:** Illustrative quotes for factors influencing parental HIV status disclosure

Parental fear of judgment	Because, you know children, they don’t understand what HIV is, some they don’t know about the confidentiality, so they will think it is just normal, so they will go telling people and they will be discriminated.—mother of four
	The child might not keep the secret, they might try to share it with other children, you see, so for me I don’t think it is important to share your status with your children. When they are playing, ‘you know my parents has this and this’, and you know they don’t have secrets at times.—mother of two
	It is important [to disclose] but at that age when they can’t understand anything and they might talk…when he/she is with her friends or her teacher, or somebody there will hear you know. It’s good to wait for that age when the child can understand everything about life that[‘s] the time now you should disclose.—mother of one
	Maybe, you know according to them HIV is usually brought about at times by other things, they will think otherwise. They might think you might have been going out of your marriage, so you might decide not to tell them.—mother of two
	They will feel that their parent[s] did not trust each other, maybe they were promiscuous.—father of three
Parental wishes to protect children from distress & child evolving emotional maturity	Because he has not matured mentally and [doesn’t] understand much, so he will feel sorry for you all the time and whenever you get sick, he/she will feel that you are going to die, and that will create a lot of worries which is not good.—mother of two
	It’s 50/50, sometimes it’s important [to disclose] and sometimes it’s not important. The kids will start like, ‘our mother is leaving us very soon’ you know they don’t understand, as you understand it. In my case as for me, I can’t tell them. No I can’t tell them, they will start thinking that I am dying tomorrow.—mother of four
	The child could get shocked because he/she might feel that maybe the parent is going to die, maybe you can even fall sick and he/she will be thinking ‘is my mum going to die from that particular disease’, it’s that kind of shock that can scare the child.—mother of three
	If the child has not matured, I will not tell him/her but I will counsel him/her and treat him/her in a way to show him/her that I love him so much so that the child doesn’t get stressed but I will not tell him/her about my status.—father of ten
	At the age of maybe 12, 13, 14 [years] I would speak to them like in a third person, you know people get sick and this and this…but when I think they get to stage like 17, 18 (years), now I think they can start to understand, say I think at that age.—mother of one
Parental planning for the future & child evolving role as caregiver within family	I think if the parents fall sick the child will know the steps to take and they will know the person to confide in.—mother of three
	Now if a child knows that you have the virus, he/she will respect you…if you tell him/her to take you to the hospital you are unwell, the child will do that because he/she respects the parents and is empathizing with the situation.—father of ten
	That is when will share my status [child aged 17 or 18], but all through, I will even be bringing them to the clinic, getting them to know, what is this that is going on, you know, but sharing my status, that is when I am saying I will share. But all through from that age, you just share small, small.—mother of one
Child evolving sexuality	When I start seeing him/her with some girlfriend or boyfriend, so maybe he will come with a girl and tell me, ‘Mum, this is my girlfriend’ you see, and the way boys are, maybe she is not the only one. Then he goes and meets another girl and I might secretly hear about it before he tells me about it, so I will tell him, ‘Yesterday you came with a different girl and I have heard there is another girl you are seeing, my son this is how the world goes, you should take care of yourself’ so that is where I will start—mother of one
	At least when you know that they are almost starting to be sexually active, then if you are [HIV positive], you can tell him, I have this and it’s not a good thing, it’s not that I am going to die but it’s not something good to have, if you can protect yourself, do it, then they can understand.—mother of two
	It is important [for them to know] if the children are grown, so that they can know how to protect themselves. And for them to know that HIV exists because most of them just hear about it, so that they can take it as something real.—mother of one

### Parental decision-making on disclosure & child’s evolving emotional maturity

Parents frequently referred to a child’s “age” to describe when they felt that a child might be emotionally mature enough to comprehend HIV transmission and disease progression without emotional distress. When children were “young” or emotionally immature, parents described strong fears and reluctance to disclose, and fear of the child’s inability to comprehend HIV transmission.

*If they are younger, they will not [understand], like now a kid who [is] 8 years, now you start telling him how you got this disease, how it is spread, how will he understand, it’s not necessarily for such very young kids… -* mother of two

Parents described plans to intentionally and gradually share certain information about their status to match their child’s maturity level.

*So you just tell them about what the disease is, you tell them about how you can manage it and all that, you prepare them so that they have the knowledge about the disease. So when the time come[s], you just tell them and I don’t think it will be such a big issue. -* mother of two

*In the future we can talk about it [my status] and I will explain to him because I will start from the earlier days, I will tell him, ‘The world is not safe, even if you find girls, just have one girlfriend, and the reason I am telling you this is because I was also this way and that… is what happened and I am not lying to you, I am telling you the truth and I have lived with this thing [HIV]…I will tell him and because he will be an adult he will understand….* - mother of one

### Child’s evolving role within a family

Some parents described the benefits of disclosing to their children. Parents described children growing older, more emotionally mature, and being able to take on new caregiving roles within a family, which increased parental perception of the benefits of disclosing their own status to a child. Parents also mentioned that if a child is old or mature enough they can provide care and support for sick parents.

*There are benefits [to disclosing], maybe for example, when you get ill, you are now very sick, the child might know how to handle you, you see, the child might know what to do and what not to do.* - mother of two

*You can disclose to one child who is mature enough to understand, because you might get sick and she is there and she is mature enough to take care of you, and she can give you medicine, she can take care of you because she knows what is going on.-mother of one.*

### Child’s evolving sexuality

Parents described a child’s sexual activity as a motivator to disclose their status to a child, as a way of educating the child to take precautions to prevent HIV acquisition.

*I think my child has the right to know what is going on, what the mother is going through and I think I would be saving his life, maybe at that time he would be thinking of having a girlfriend, maybe he would be having the thought of having sex, but because I have said, I have talked to him and I have told him what I am going through, he will have a second thought…He would think, ‘ok fine, I am going but I will use protection’.-mother of one.*

### Parental fear of judgement for own status by the community and their children

Parents who felt shame, embarrassment, or fear of judgement related to their own HIV status expressed unwillingness to disclose their status to their children. While the higher-level theme was the child’s maturity level to understand what living with HIV means, parents were also concerned that a child might be too young to understand confidentiality or how to “keep secrets”, and the child revealing the parent’s HIV status to others in the community would result in parental emotional distress.

*You know if I tell them…they are going to tell others and when they tell others out there, people will start talking about me, you know when people are talking about you out there, it will come back to me and you know I will start getting stressed.* - father of ten

Some parents were reluctant to disclose their status to their children out of concern that it would negatively affect the child-parent emotional bond.

*They might think you might have been going out of your marriage, so you might decide not to tell them*. - mother of two

*In my opinion, you will create tension, you will create a gap…there is no sense of telling them that I am HIV positive… when I start telling them that I am HIV positive, that faith that they had in me can be affected.* - father of two

Some parents however felt that disclosure to children would improve respect.

*The baggage will be off now, there will be a load off now…Because I think he will even respect me more knowing that I have shared with him something so personal.* - mother of one

### Parental wishes to protect children from distress

Parents frequently spoke about wanting to protect children, particularly younger children, from distressing knowledge or thoughts. They voiced concerns that younger children who learned of their parent’s HIV status would fear parental death, or that knowing their parent’s status would cause the child stress and anxiety.

*Sometimes it not so fair because you might make them to lose hope of their life, if they are schooling, they might think that our parent is dying before we finish our education. You know sometimes some children think that if you have HIV you might die the next minute. It might kill their hopes.* - mother of one

### Parents’ wish for support during disclosure

While reporting their willingness to disclose their HIV status to their children in the future, parents requested tools to assist them in the process.

*I would like, maybe if I could be given picture[s]…you know with the pictures, if I go back to the house, I could take the place of the picture and I could explain to the child using the pictures yet be talking about myself. - father of two*

## Discussion

Parental disclosure of their HIV status to their children was infrequent. Most caregivers included in the study had known their HIV status for at least a year and were already engaged in HIV care for themselves. Not surprisingly, increase parental and child age increased the likelihood of disclosure. Our qualitative data suggests that parental readiness to disclose is influenced by barriers such as anticipated stigma from children and the community, and facilitators including increasing child maturity, their evolving roles in the family and sexuality, and the parent’s belief in the right of the child to know their parent’s HIV status. Parental readiness was tightly linked to their perceptions of their child’s readiness. Parental wishes to protect children from emotional distress were often tied to descriptions of the child’s lack of emotional maturity. Parental plans for their own future care often followed ideas of a child growing into additional caretaking roles within the family. However, parental fear of judgement for their own status and their child’s emerging sexuality were discussed relatively independently. Finally, parents, while expressing willingness to disclose in the future, pointed out a need for assistance in disclosing.

The prevalence of parental disclosure reported in our study (13%) was on the lower end of the wide range of prevalences reported previously (11–50%). Previous studies have noted that disclosure to children may be prompted by child HIV testing [34]. Because the inclusion criteria for this study required that participants have at least one untested child, the population selected for this study may thus have had lower levels of parental disclosure than those found elsewhere. However, we found that the majority of participants had disclosed to someone (85%). This was unsurprising since most of the caregivers enrolled in this study were in stable relationships with partners living with HIV. The failure to disclose to their children, however, could be due to their having very young families (median age of oldest child being 11 years). Typically, in sub-Saharan Africa, children living with HIV have their own HIV status disclosed to them at a median age of 13 years [35–37]. Assuming that the age of parental disclosure follows this pattern, the very low prevalence of parental HIV status disclosure to children in our study is unsurprising.

The qualitative findings of this study enabled us to understand factors that act as barriers and facilitators to support parental disclosure. At an individual level, all participants noted the child’s age or perceived maturity as a key factor to consider before disclosure. The theme of child’s age influencing parental disclosure was also prominent in the quantitative data. We found that having older children, which in our study was determined by the age of the oldest child, was a significant factor associated with parental disclosure. In the qualitative data, the influence of the child’s age came across in various ways: parents feared that their young children would not comprehend HIV transmission, that the children would be stressed or worried about losing their parents or that the child would not be able to keep their parent’s HIV status secret, resulting in inadvertent disclosure. These fears are commonly reported in the literature as influencing factors of whether a parent will disclose their status to their children [19, 21, 34, 38, 39]. Previous studies that have interviewed parents and children after parental disclosure have mixed findings; while some studies report that the proportion of children with negative reactions can be up to 50%, the majority of studies report that children cope well with the disclosure in the long term. A few studies show that after parental disclosure, children develop externalizing behaviors and lower social competence [5, 8, 38]. Additionally, one study in Haiti confirms the fears mentioned by parents we interviewed that the child will be worried and upset following parental disclosure [40]. Other studies in Burkina Faso and Uganda, however, found that many children knew or suspected their parent’s HIV status before the disclosure, did not disclose the information to others, were not blamed, and the children became treatment supporters [40–42].

The fears expressed by the parents could also be viewed as a struggle to define the “right age” to disclose. In previous studies, parents commonly stated that they would disclose once their children were older without specifically defining the “right age” [5, 19, 43]. The quantitative results also showed that older child and caregiver age predicted a higher likelihood of parental disclosure. Further, the child’s evolving sexual maturity was found to play a facilitating role in the willingness of the parent to self-disclose. Parents described a desire to share their own experiences in the hope that their children would learn from them and protect themselves from HIV infection through safer sexual behaviors. While guidelines propose that parental disclosure be done at a similar age to disclosure of the child’s own HIV status [29], there is need for the development and scaling up of tools to support caregivers in the self-disclosure process [22]. The request for disclosure tools by parents in this study reflects their inability or lack of confidence to frame their HIV status in a way that they believed their child could understand. Similar to the disclosure of the child’s own HIV status, parental HIV status self-disclosure may be included in a broader package that equips children living with HIV or from households with parents living with HIV with reproductive health literacy. [44].

All but one of the interviewees discussed their plans to disclose to their children in the future. In discussing their plans for disclosure, several participants felt they would benefit from assistance in the process, including having tools with graphics describing HIV. Potential interventions to support parents through disclosing to their children include targeted professional counseling, peer counseling or support groups, providing a venue for assisted disclosure [43, 45] and equipping parents with appropriate techniques for disclosure. Lack of appropriate disclosure techniques has been highlighted as a key barrier for parental disclosure in previous studies [46, 47]. The tools and the self-disclosure process should provide safety nets to deal with the parents’ concerns of child distress and anxiety resulting from the disclosure. Examples of interventions that can be adopted in our setting include the Amagugu from South Africa, [25] and others developed in Rwanda [11] and in high-income settings [23].

Further, parental HIV infection is a source of stigma for parents and children [48, 49]. For parents, this includes anticipated stigma from their own children expressed as fear of judgement by children and children losing faith in them. The process of parents sharing their HIV status with their children, if properly supported, could bolster anti-HIV stigma efforts among children and parents [45, 50]. Additionally, community-level anti-stigma interventions, including those targeting children, especially in school [51], may improve the children’s attitudes towards people living with HIV and provide a good environment for parental disclosure.

The majority of parents in this study voiced the firm belief that the child “had a right to know” the parent’s status as a motivation for eventual disclosure, which was echoed in previous studies [19]. This belief could come from the parent’s ethical concerns over what is the right thing to do or a more practical desire to ensure that their children knew of the parent’s status in the event that they needed care or support from their children or if the parent were to pass away [34]. Though not studied, parental disclosure may also be relevant when discussing some of the challenges HIV exposed uninfected children may be facing, including neurocognitive delays [52, 53].

Strengths of our study include the use of a mixed methods approach that included in-depth interviews and surveys to understand rates, and barriers and facilitators of parental HIV disclosure in Kenya. However, the quantitative study had more diversity with participants recruited from different facilities and regions in Kenya whereas the qualitative study involved participants from only one site. Additionally, participants were selected from among those enrolled in an HIV treatment program with at least one untested child (12 years or younger), and these individuals may differ from those who are not enrolled in care or those in care with only older children. The study also underrepresented views of parents who were male due to the parent study’s eligibility requirement for the consent of the mother of the untested children targeted for index case testing. This extra step in enrollment for fathers resulted in a moderately skewed study population in favor of mothers. Additionally, we could not observe incident disclosure and study the evolution of barriers or facilitators of disclosure especially with increasing child age due to the cross sectional design of our study. Further, we did not establish the relationship between parental disclosure and key child characteristics such as age and education since we did not collect parental disclosure data that was specific to any one child a caregiver had. The age of the oldest child, however, provided some indication of how old the children whom the parent could have potentially disclosed to were. Lastly, while we felt that the combination of primarily deductive coding plus inductive for newly emerging themes allowed us to reach thematic saturation, we did not assess for meaning saturation, which is a limitation of the present qualitative analysis.

## Conclusions

Parents living with HIV were open to disclosing their HIV status to their children. There is a need for interventions to remove some of the barriers that have resulted in a low prevalence of disclosure, including anticipated stigma from their children and the community, and fear that disclosure will cause distress to their children. While implementing guidelines on parental disclosure, providers should address these parental concerns and take into consideration the factors that may influence the optimal age of the child for disclosure, including the child’s maturity, role in the family and sexuality. Tools for parental disclosure as well as provision of peer and provider support are urgently needed to support the disclosure process.

## Data Availability

The data used in this analysis are not publicly available but are available from the corresponding author upon reasonable request.
